# An empirical study on cultural identity measurement and its influence mechanism among heritage tourists

**DOI:** 10.3389/fpsyg.2022.1032672

**Published:** 2023-01-20

**Authors:** Yuanbo Fu, Jian Ming Luo

**Affiliations:** ^1^School of Tourism, Ningbo City College of Vocational Technology, Ningbo, China; ^2^Faculty of International Tourism and Management, City University of Macau, Macau, China

**Keywords:** cultural heritage tourism, cultural identity, measurement, influence mechanism, empirical study

## Abstract

In the effort to integrate culture and tourism, an important strategy for the prosperity and sustainability of tourism, it is necessary to factor in tourists’ cultural identity and emotional interaction with cultural heritage to keep pace with the trends of cultural heritage tourism. Drawing on in-depth interviews and software such as Nvivo and Smart-PLS, this study aims to develop and verify cultural identity measures for cultural heritage tourists, construct a theoretical model of cultural identity, travel experience, place attachment, satisfaction, and revisit intention, and verify the theoretical relationship between these dimensions in the Archaeological Ruins of Liangzhu City. The results show that cultural identity is positively associated with travel experience and place attachment but not significantly associated with satisfaction; travel experience is positively associated with place attachment and revisit intention; place attachment is positively associated with satisfaction; place attachment is positively associated with revisit intention; satisfaction is positively associated with revisit intention. This qualitative and quantitative research enriches the theoretical achievements concerning cultural identity among cultural heritage tourists and proposes recommendations for management practice accordingly.

## 1. Introduction

As an integral part of China’s excellent traditional culture, cultural heritage has become an important tourism resource, nourishing the people spiritually. Tourism provides an essential way for direct contact and interaction between cultural heritage and the people and becomes an essential means of exercising the social and cultural value of cultural heritage (Luo and Ren, 2020). With the transformation and upgrading of the tourism sector, the cultural taste of tourists has been continuously improved, and the industrial status of cultural heritage tourism has become increasingly prominent. The integration of cultural heritage and tourism has become an important strategy for a country to promote the development of cultural undertakings and realize the prosperity and sustainability of tourism (McKercher et al., 2002; Luo, 2022). In *The 14^th^ Five-Year Plan for National Economic and Social Development and Outline of Vision for 2035* issued in March 2021, there are a number of contents pertaining to the cultural tourism industry, which provides a favorable policy environment for the development of cultural heritage tourism.

Since the end of the 20^th^ century, the research on heritage tourism has touched upon the basic theory, interpretation, development, and marketing of heritage tourism, the authenticity and influence of heritage, etc. The related explorations of cultural identity in tourism can be classified into two categories. The first category tries to explore the path and method of enhancing cultural identity through tourism using cultural identity as a dependent variable. The second category attempts to examine the influence of cultural identity on related variables using cultural identity as an independent variable. In general, the existing research mostly discusses the enhancement of cultural identity through tourism, but little attention is paid to how cultural identity affects tourists’ psychological perception and behavioral intentions, and even less research exists in the field of cultural heritage tourism. Entering the new era, how to enable cultural heritage to meet the people’s growing needs for a better life and become a cultural link interacting with the masses has become a concern. Tourists, as the subjects of cultural heritage tourism, have an impact on the protection and development of cultural heritage. Therefore, in the effort to integrate culture and tourism, it is necessary to probe into tourists’ cultural identity and emotional interaction with cultural heritage to keep abreast with the trends of cultural heritage tourism. What is the cultural identity of tourists to the corresponding cultural heritage? How does this shape their travel experience and place attachment during the travel? What impact does it have on their satisfaction and revisit intention? What implications does this have for the development and management of cultural heritage tourism? The present study tries to explore the theoretical relationship among heritage tourists’ cultural identity, travel experience, place attachment, and satisfaction through an empirical analysis of the Archaeological Ruins of Liangzhu City, a world heritage site.

## 2. Literature review and research hypotheses

### 2.1. Cultural identity

The research on cultural identity mainly concerns national cultural identity, residents’ identity with tourism culture, and tourists’ identity with the culture of tourist destinations in fields such as marketing and tourism. Many studies in the field of marketing have shown that cultural identity significantly affects consumers’ purchasing behavior. In the process of consumption, people build a sense of self and psychological identity through the use of symbols (Belk, 1978). After an individual’s cultural identity is generated, the corresponding willingness to consume will also be generated. To reflect their own values, consumers will consume products with the same or similar cultural background (Friedman, 1994). The higher the degree of consumer identification with the non-home culture, the stronger their willingness to buy exotic products, but there are some interference factors (Yong and Zhina, 2008). An empirical study on Hip-Hop exhibited that the stronger an individual’s cultural identity, the more positive attitude she or he will have towards the brand (Ferguson and Burkhalter, 2015). On the contrary, another study reveals that consumers’ cultural identity does not have a direct positive impact on purchasing behavior, and the brand’s local symbolic value plays a complete mediating role between the two (Haiyang and Jiaxun, 2017).

The antecedents and mechanisms of cultural identity have also been investigated in the field of tourism. For African Americans and Caucasians, feelings about the heritage and expectations for learning at the heritage site are important factors encouraging them to travel to Cape Coast Castle in Ghana (Austin, 2002). Cultural identity is one of the motivational factors for tourists to visit ex-Yugoslav heritage in Croatia (Goulding and Domic, 2009). Park believes that heritage tourism is not only the tourism consumption behavior of heritage and cultural relics, but also the reconfirmation of national significance and value (Yu Park, 2010). Cultural representation is based on cultural identity, and the content, form, and influence of cultural representation depend to some extent on the degree of the cultural identity of local residents (Xingfu and Lin, 2014). Tourists’ recognition of the tourist area’s culture can enhance their place attachment and revisit behavior, and the intangible cultural elements of the tourist area are also an important factor in forming tourists’ emotional connection to the tourist area (An'na, 2015). Tourist involvement and cultural identity have a positive impact on the tourists’ destination image; this image has a positive guiding effect on tourists’ revisit tendency, but tourist involvement and cultural identity have no direct impact on revisit tendency; the image of the tourist destination plays a complete mediating role among tourist involvement, cultural identity, and revisit tendency (Xiangyang et al., 2015). Carol and others emphasized that heritage tourism is the main medium to tell national stories on behalf of collective memory and enhance social cohesion by shaping the uniqueness of a country (Zhang et al., 2018).Yang et al. believed that the rational utilization and effective protection of historical and cultural resources serve not only as an important path for the modern tourism industry to promote cultural identity while respecting tourists’ cultural differences but also as a cornerstone for the rapid and stable development of the tourism industry in western China (Yang and Jiang, 2020).

It can be found that most scholars have studied cultural identity as an independent variable, mainly examining the impact of cultural identity on an individual’s travel decisions and behavioral intentions. In contrast, some researchers tend to use cultural identity as a dependent variable to explore ways to enhance cultural identity. At present, there is scant research on cultural identity among heritage tourists. For this reason, this study attempts to construct a theoretical model with cultural identity as an independent variable and verify its impact mechanism through an empirical case study.

### 2.2. Travel experience

The research on travel experience began in the 1960s. Boorstin believed that there is a difference between tourists and travelers; mass tourists are often misled by false events; the travel experience is predetermined, rigid, and contrived (Boorstin, 1964). Cohen classified travel experience into diversion, experimentation, existence, entertainment, and experience and posited that different tourists have different needs for travel experience (Cohen, 1979). Ryan analyzed the commonality of travel experience and pointed out that travel experience is a multi-functional leisure activity aimed at individuals, involving entertainment or learning, and it is an inner feeling formed by tourists participating in tourism activities (Ryan and Crotts, 1997). Pine and Gilmore classified travel experience into educational experience, escape experience, entertainment experience, and aesthetic experience based on a matrix with consumers’ participation as the horizontal axis and consumers’ association with their environment as the vertical axis and developed a 4E scale for measuring experience (Pine and Gilmore, 1998). Stamboulis and Skayannis argued that the richer the tourists’ experience activities, the more profound the tourism perception (Stamboulis and Skayannis 2003). Ballantyne et al. proposed that tourists’ cognition and behavior in traveling largely depend on their experience (Ballantyne et al., 2011). In the late 1990s, Xie suggested that the key to tourism research lies in travel experience, which refers to a kind of physical and mental experience and emotional cognition obtained by tourists who deeply integrate with the situation during travel (Xie, 1999). Stamboulis and Skayannis consider the travel experience as an interaction between the visitor and the destination (Stamboulis and Skayannis 2003). Larsen defines a travel experience as a past travel experience that is important enough to be remembered for a long time(Larsen, 2007).Cultural experience plays an important role in the development of cultural heritage tourism(Dredge, 2004),which is a key component of travel experience in cultural heritage destination. Some studies focus on the travel experience of cultural heritage(Artal-Tur et al., 2018). Cetin and Bilgihan proposed a five-dimensional construct of travel experience in a World Heritage Site(Cetin and Bilgihan, 2016). Seyfi et al. investigated travel experience in a heritage site and highlighted six components (Seyfi et al., 2020). With the development of tourism, tourists’ requirements for experience have gradually improved. They desire to experience various natural and humanistic elements in tourist destinations so as to have travel experiences that are different from daily life (Xu et al., 2022).

This line of evidence suggests that the formation of the travel experience is inseparable from a specific situation. Travel experience highlights the experience at the psychological level, and the result of the travel experience is manifested in psychological pleasure. This paper argues that the travel experience is a process in which tourists have a temporary connection with the outside world through participating in tourism activities, and on this basis, they change or adjust their psychological level or psychological structure.

### 2.3. Place attachment

The concept of place attachment emerged in the late 1950s when some phenomenologists abroad discovered that people would endow some specific places with psychological significance. Eliade revealed that places of worship have special significance for people (Eliade, 1959), and Bachelard believed that the home is a space that symbolizes happiness, shelter, and imagination (Bachelard, 1987). In the late 1970s, the term place attachment appeared formally and was incorporated into the system of environmental behavior, which brought a strong impetus to scholarly research in this regard. For the past 50 years, there has been a growing interest in the human-land relationship (Di Masso et al., 2019), which is widely known as place attachment (Ariccio et al., 2020). The Guttman scale and the Likert scale are the most frequently used among the manifold place attachment scales. The former is generally used to measure the place attachment level of local residents, while the two-dimensional Likert scale consisting of 27 measurement questions in place identity and place dependence is mainly used to analyze tourists’ degree of attachment to the tourist destination(Williams et al., 1992). In a different perspective, researchers have included affective and social components in the study of place attachment. Jorgensen and Stedman conceptualized affective attachment as an emotional bond with a particular setting(Jorgensen and Stedman, 2001). Based on the previous efforts, Williams et al. examined the validity and generalizability of place attachment across measurement items, places, and dimensions of place attachment (Williams and Vaske, 2003). This study mainly refers to Williams’ scale and makes appropriate adjustments according to the specific research situation.

### 2.4. Tourist satisfaction

With the development and rise of tourism, tourist satisfaction has drawn more and more scholarly attention. Dorfman argued that tourist satisfaction depends on both subjective and objective factors: the subjective factors come from the tourists themselves, while the objective factors are mainly related to the tourist destination (Dorfman, 1979). Baker and Crompton defined tourist satisfaction as the real psychological perception after a variety of tourism activities and travel experiences, which is the comprehensive evaluation of tourists on all tourism activities at the overall level (Baker and Crompton, 2000). The concept of tourist satisfaction originated from the field of marketing and is the embodiment of consumer satisfaction in the tourism sector. Tourist satisfaction is the real feeling of tourists after participating in tourism activities, and the overall evaluation of to the whole trip. The SERVQUAL model (Zeithaml et al., 1988) was later widely recognized by academia as a classic method for evaluating service quality. Some researchers took the historical district of Istanbul as the research object and developed a three-dimensional structural measurement scale (Altunel and Erkurt, 2015). Other scholars measured tourist satisfaction using two questions for heritage attractions (Huete-Alcocer et al., 2019). In the tourism literature, various studies were conducted to research the relationship between tourist satisfaction and post-purchase intentions (Harrison and Shaw, 2004; Hui et al., 2007; Theodorakis et al., 2013). Tourist satisfaction is a major factor in generating positive impressions, attracting more visitors, and increasing visitors’ loyalty (Yi et al., 2018). The above research provides a good foundation for the study of the measurement and influence mechanism of cultural heritage tourist satisfaction.

### 2.5. Revisit intention

Drawing on the repurchase intention from the field of marketing, Gyte and Phelps investigated the behavior of British tourists after visiting Spain and found that they had a strong intention to revisit tourist destinations (Gyte and Phelps, 1989). Scholars developed a two-dimensional scale to measure tourists’ revisit intention in terms of behavioral loyalty and attitudinal loyalty (Backman and Crompton, 1991; Dick and Basu, 1994). The revisit intention is often subject to factors such as tourists’ own travel experience, traffic conditions, local price levels, and public attitudes (Kozak, 2001). Jang et al. classified tourists’ revisit intention within 5 years into persistent revisit intention, delayed revisit intention, and persistent purchase intention (Jang and Feng, 2007). Assaker and Hallak conducted a time-based study on revisit intention according to the length of the time interval between tourists’ first visit and their revisit intention (Assaker and Hallak, 2013). The derivative meaning of revisit intention also includes “recommendation intention,” that is, the willingness to recommend relatives and friends to travel to the destination (Guo, 2016). Andrades and Dimanche found that perceived value significantly influences the satisfaction of tourists, which in turn has a significant and positive impact on tourist loyalty(Andrades and Dimanche, 2018). Cevik utilized the structural equation model (SEM) to empirically analyze the relationship between park satisfaction, place attachment, and willingness to revisit the old place and measured the revisit intention using three questions (Cevik, 2020). According to the objectives of this study, the revisit intention is defined as the psychological tendency of tourists who are willing to pay another visit and recommend others to travel after a tourist experience in a certain destination.

### 2.6. Research hypotheses and modeling

#### 2.6.1. The relationship between travel experience, place attachment, and tourist satisfaction

There is scant research on the impact of cultural identity on the travel experience of the destination. Catherine Palme believes that the reason why heritage tourism is favored by tourists is that the historical and cultural symbols of heritage sites are recognized by tourists, which not only attracts tourists, but also enhances the image of heritage sites(Palmer, 1999). Cultural tourism has recently been re-affirmed by the UNWTO as a major element of international tourism consumption(Richards, 2018).An experimental study where cultural identity was divided into transient active and stable cultural identity demonstrated that stable cultural identity has an impact on the perceived value of tourist destinations (Xue, 2014). Studies by Jameson and Gomez et al. have shown that consumers have a higher opinion of products that are consistent with their own cultural identity(Jameson, 2007; Gomez and Torelli, 2015). According to Arkas’s research, customers will consider product quality according to their own opinions, and consumers’ product cognition will positively affect the perceived quality(Viddy et al. 2018). Rust et al. show that consumers’ emotions affect perceived quality(Rust et al., 1999).It follows that that the stronger tourists’ cultural identity with the destination, the higher the perceived value of the destination, and the more positive the evaluation. Therefore, this study proposes the following hypotheses:

*Hypothesis 1*: Cultural identity is positively associated with travel experience.

The emotional relationship between people and places is complex, and people have a natural sense of attachment to the environment. This emotion sometimes is called topophilia, namely, “the emotional connection between people and places or environments, or people’s love for places” (Song, 2012). Through empirical research, Li found that tourists’ cultural identity to tourist areas can enhance their place attachment (Yang and Jiang, 2020). For cultural heritage sites, culture is the core tourism resource, and some places often serve as the carrier of a specific culture and become the symbol and representative of this culture. Tourists with a strong cultural identity may develop a deep sense of attachment and belonging to the tourist destination. Based on the above analysis, this study proposes the following hypothesis:

*Hypothesis 2*: Cultural identity is positively associated with place attachment.

Few studies have directly touched upon the relationship between cultural identity and tourist satisfaction. According to the definition given hereinbefore, the cultural identity reflects positive emotions towards a specific culture. A survey of Spanish tourists’ satisfaction confirmed that tourists’ positive emotions have a significant impact on satisfaction (Bosque and Martín, 2008). An empirical study on the satisfaction of tourists traveling to Thailand also showed that positive emotions have a significant positive impact on satisfaction (Hosany et al., 2016). Hence, this study proposes the following hypothesis:

*Hypothesis 3*: Cultural identity has a significant is positively associated with tourist satisfaction.

#### 2.6.2. The relationship between travel experience, place attachment, and revisit intention

The research on cultural heritage travel experience and place attachment has made some progress but is still to be deepened. Suntikul and Jachna explored the relationship between tourists’ experience of a World Heritage Site and place attachment. In their study, experiential value is based on Pine and Gilmore’s four domains of experience (entertainment, education, aesthetics, and escape; Pine and Gilmore, 1998), while the concept of place attachment is defined based on place dependence and place identity (Williams and Vaske, 2003). They found that travel experience has different impacts on each dimension of place attachment (Suntikul and Jachna, 2016). Du et al. confirmed that emotional experience has a positive impact on place attachment after their exploration of the relationship between tourism authenticity, emotional experience, and place attachment using SEM (Du et al., 2018). This line of evidence indicates that the travel experience in cultural heritage sites is likely to affect tourists’ level of place attachment. Therefore, this study proposes the following hypothesis:

*Hypothesis 4*: Travel experience is positively associated with place attachment.

Sufficient attention has been paid to the relationship between travel experience and tourist satisfaction. Judging from the definition of tourist satisfaction, tourists’ satisfaction is their comprehensive evaluation and overall feeling of the tourism process (Baker and Crompton, 2000). Mano and Oliver postulated that there is a positive correlation between customer experience and satisfaction (Mano and Oliver, 1993). To a certain extent, the satisfaction of tourists is the satisfaction with the travel experience. Improving tourists’ experience can greatly improve their overall evaluation of the scenic spot. When the travel experience can meet the needs of tourists and create a pleasant feeling for them, their satisfaction will be higher. By improving tourists’ experience, their satisfaction can be increased. An empirical exploration of self-portrait tourism exhibited a significant positive correlation between memorable travel experiences, hedonic well-being, and place attachment (Li et al., 2021). It is safe to infer that the travel experience of tourists in cultural heritage sites is likely to affect their level of place attachment. Therefore, the following hypothesis is put forward:

*Hypothesis 5*: Travel experience is positively associated with tourist satisfaction.

Extensive research has been conducted on the relationship between experience quality and post-purchase behavior or behavioral intentions in the field of consumer behavior. The better the overall experience of consumers, the more positive the behavioral response after the experience (Schmitt, 1999). Consumers’ perceived experience when shopping in stores will directly affect their enjoyment experience and will further determine their subsequent shopping behaviors (Ibrahim and Wee, 2002). In the field of tourism, travel experience plays an important role. Tourists who are satisfied with the travel experience will have a higher degree of loyalty and are more likely to revisit, which has been confirmed by various scholars. Improving the quality of tourists’ experience can not only enhance their willingness to revisit but also bring favorable publicity to scenic spots (Gitelson and Crompton, 1984). In an empirical study on Caribbean tourists, their satisfaction experience has a remarkable positive impact on revisit intention (Petrick, 2004). Another study showed that all dimensions of unforgettable travel experience are predictors of tourist satisfaction (Tsai et al., 2021). Hence, this study proposes the following hypothesis:

*Hypothesis 6*: Travel experience is positively associated with revisit intention.

#### 2.6.3. The relationship between place attachment and revisit intention

The concept of place attachment originated from environmental psychology, which refers to an individual’s sense of belonging to a specific place (or site; Bricker and Kerstetter, 2000). Later, it became an important concept in the fields of human geography and environmental studies to describe the emotional link between people and places (Tang, 2007). In the field of tourism, place attachment refers to the emotional connection between tourists and the destination in the process of interaction. The stronger the sense of belonging and identity of tourists to the destination, the more likely they will make a positive evaluation of the destination (Yuksel et al., 2010). The COVID-19 pandemic has dynamically reconfigured people’s connection to places and restored people’s sensitivity to places (Devine-Wright et al., 2020). Emotions between people and places play an important role in shaping tourists’ satisfaction, and place attachment have a positive impact on satisfaction (Ramkissoon, 2013). Studies have established that tourists’ place attachment has a positive effect on satisfaction. Therefore, this paper proposes the following hypothesis:

*Hypothesis 7*: Place attachment is positively associated with tourist satisfaction.

The literature review shows that scholars in the field of tourism often regard place attachment as the emotional link between tourists and the destination and analyze its influence on tourists’ behavioral intentions. A study on the influencing factors of tourist loyalty in Xiamen, Fujian Province, illuminated that place attachment is the most favorable force for tourist destinations, which has a positive impact on tourists’ behavioral intentions and encourages tourists to revisit and recommend others to visit tourist destinations (Jia and Lin, 2016). The stronger the tourists’ place attachment, the higher their behavioral intentions, and the more likely they will recommend and share the destinations (Kyle et al., 2005). Hence, this study proposes the following hypothesis:

*Hypothesis 8*: Place attachment is positively associated with revisit intention.

#### 2.6.4. The relationship between tourist satisfaction and revisit intention

Tourist satisfaction is widely used as an indicator of revisit intention, and many scholars have confirmed that revisit intention is associated with tourist satisfaction (He and Luo, 2020). Among Istanbul cultural tourism enthusiasts, satisfaction, tourist participation, and experience quality jointly affect tourists’ revisit intention (Altunel and Erkurt, 2015). For tourists in Alishan National Forest Recreation Area, their satisfaction has a significant positive impact on revisit intention (Sadat and Chang, 2016). Cevik used SEM to empirically analyze the relationship between park satisfaction, place attachment, and willingness to revisit the old place (Cevik, 2020). Other researchers also found that tourist satisfaction is the most important factor affecting loyalty (Ajayi and Tichaawa, 2021). Therefore, this study proposes the following hypothesis:

*Hypothesis 9*: Tourist satisfaction is positively associated with revisit intention.

**Figure 1:**
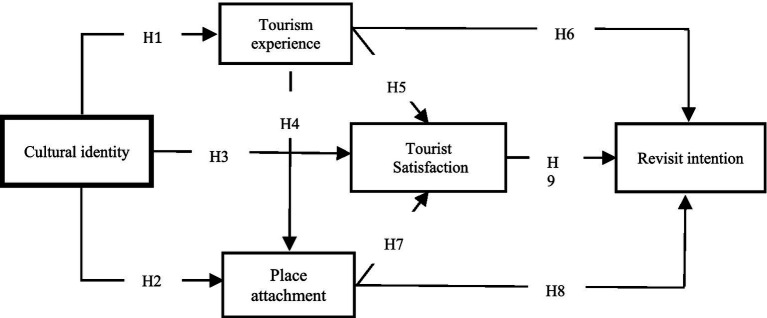
Conceptual model.

Based on the concepts mentioned above and the possible relationships between them, this study designs a corresponding conceptual model (Figure 1) and proposes nine research hypotheses.

## 3. Methods

### 3.1. Measuring instrument

We formulated the interview outline and pre-tested the preliminary questionnaire with reference to the existing scales of cultural identity, travel experience, place attachment, tourist satisfaction, and revisit intention pertinent to cultural heritage tourism. The questionnaire consisted of six parts, namely, cultural identity, travel experience, place attachment, tourist satisfaction, revisit intention, and the personal information of the respondents. According to Lietz (2010), the 7-point Likert scale is more reliable and discriminating than the 5-point one. Therefore, the core part of the questionnaire used in this study was scored by the 7-point Likert scale (1 = inaccurate at all; 7 = fully accurate).

A cultural identity is essentially a form of collective identity that binds people together based on a shared history and cultural heritage, reflecting people’s positive feelings about a culture. The case under this study is the Archaeological Ruins of Liangzhu City, which features both abstract cultural connotations and tangible cultural carriers. Therefore, it is necessary to transform the identity of Liangzhu culture into easy-to-understand and quantifiable sentences and modify the corresponding cultural identity scale. Retrieving relevant studies using keywords such as cultural heritage and cultural identity and referring to previous studies (Phinney 1992; Wang and Chen, 2016; Pan et al., 2019; Tian et al., 2020), we built a preliminary cultural identity scale composed of 12 questions in terms of cognition, emotion, and behavior, which was further modified through data analysis and feedback by questionnaire pre-test, in-depth interviews, pre-investigation (Table 1).

**Table 1:** The cultural identity scale used in the present study.

**Dimension**	**Question**	**Reference**
Cognitive	I know the period of the cultural heritage.	Phinney (1992); Lee and Yoo (2004); Wang and Chen (2016)
	I know the place of the cultural heritage.	
	I know the cultural value of cultural heritage.	
	I know the cultural relics of the cultural heritage.	
Emotional	The culture of the heritage can make me feel proud.	Phinney (1992); Lee and Yoo (2004); Ferguson and Burkhalter (2015)
	I am deeply attached to the culture of the heritage.	
	I am keenly interested in the culture of the heritage.	
	We should attach importance to inheriting and developing the culture of the heritage.	
Behavioral	I often discuss matters related to the heritage with others.	Phinney (1992); Lee and Yoo (2004); Wang and Chen (2016)
	I will spend time learning about the heritage.	
	I will participate in cultural tourism activities related to heritage.	
	I will buy cultural tourism products related to heritage.	

This study adopted a mixed method. Since the scale used in this study was modified to cater to cultural heritage, to ensure that it is normative and appropriate, we conducted key interviews on the dimensions of cultural identity. The interviewees included developers, managers, and tourists of the Archaeological Ruins of Liangzhu City. The interviews mainly involved inquiries on core aspects such as cultural identity, travel experience, and their influence mechanisms, as well as recommendations for the protection, development, and management of the cultural heritage. Totally 22 online interviews were conducted from November 29 to December 1, 2021. The interview scripts were transcribed, sorted, and summarized using Nvivo for word frequency and opinion analysis. The purpose of this procedure was to verify and deepen the data obtained by the quantitative research and provide a reference for the design and management of the questionnaire. The in-depth interviews validated tourists’ cultural identity in terms of cognition, emotion, and behavior. The specific results are shown in Table 2.

**Table 2:** The qualitative analysis of cultural identity factors.

**Dimension**	**Keyword**	**Number of mentions**
Cognitive	Cultural evidence	22
	Early stage	17
	History of civilization	16
	Belief	14
Emotional	Proud	16
	Inheritance	11
	Amazing	10
Behavioral	Tourism activity	16
	Visit	15
	Interaction	10

The questionnaires were revised and adjusted based on the in-depth interviews and expert opinions. In this study, 10 experts in cultural heritage tourism were invited to review the preliminary questionnaire so as to improve the measurement accuracy of cultural identity, travel experience, place attachment, tourist satisfaction, and revisit intention. Considering the discriminant validity with adjacent questions, we deleted “I know the cultural relics of the cultural heritage” and “I am keenly interested in the culture of the heritage.” To avoid the interference of semantic emphasis on the respondents, we rephrased “I am deeply attached to the culture of the heritage” into “I like the culture of the heritage.” The remaining questions were numbered C1-C10.

The travel experience scale for heritage tourists was based on the 4E experience scale developed by Pine and Gilmore (Pine and Gilmore, 1998)and previous studies (Wei and Wei, 2004; Piera et al., 2017). The scale is divided into four dimensions: educational experience, aesthetic experience, entertainment experience and escape experience, with a total of 12 items. The place attachment scale referred to the most pertinent questions in Williams’ version (Williams and Vaske, 2003), which divided place attachment into two dimensions: “place dependence” and “place identity,” and 6 items closely related to this study are selected. The tourist satisfaction scale was derived from questions previously described (Huete-Alcocer et al., 2019)，regardless of dimensions, a total of 3 items. The revisit intention scale referred to previous studies (Dick and Basu, 1994; Guo, 2016) and concerned tourists’ willingness to revisit the Archaeological Ruins of Liangzhu City, as well as their willingness to share their travel experience with other tourists，with a total of 3 items.

### 3.2. Study case and data collection

The equations should be inserted in editable format from the equation editor. The Archaeological Ruins of Liangzhu City is located in Yuhang District, Hangzhou City, Zhejiang Province. It covers an area of 3 square kilometers and was built in 3300 BC. Liangzhu Culture belongs to the late Neolithic Age，which was 5,300–4,300 years ago，and lasted for 1,000 years. It was the power and belief center of an early country and has been known as the first city in China. On July 6, 2019, the Archaeological Ruins of Liangzhu City were approved to be included in the World Heritage List. In May 2020, the ruins were selected as the first batch of “Zhejiang Cultural Imprints.” In less than half a year, the heritage had entertained more than 1 million tourists and become a new “social media influencer.”

The respondents were selected by random sampling on site. The respondents were required to answer the questions by scanning a QR code using WeChat, which was supplemented by paper questionnaires. The pre-investigation was conducted on December 4–5, 2021, and a total of 235 questionnaires were collected, of which 200 were valid after they were screened according to the answering time, quality, and other factors. The formal survey was performed random sampling on site from December 11 to 19, 2021, and a total of 461 valid questionnaires were collected. Among the respondents, 30.6% were male, and 69.4% were female; 20.2% were aged 18–25 years, 41.2% were aged 26–30 years, and 29.7% were aged 31–40 years; most of them (70.3%) earned a monthly income of 5,000–15,000 yuan, accounting for 70.3%; their educational background was mainly undergraduate and junior college, accounting for 91.1%; most of them (79.6%) were first-time or second-time tourists.

## 4. Results

In the first stage, exploratory factor analysis (EFA) was conducted based on the questionnaire pre-test to explore the validity and reliability of each dimension of the questionnaire. The second stage was a formal survey and data analysis. The data obtained during the formal survey was first sorted out for descriptive statistics and data screening using SPSS 26.0. Then, SEM and Smart-PLS were employed to test the measurement model and structural model: (1) confirmatory factor analysis (CFA) was performed to test the validity and reliability of the dimensions of the measurement model; (2) the structural model was evaluated to examine the path coefficients, test the relationship and strength between the dimensions, and validate research hypotheses.

### 4.1. Measurement model evaluation

After pre-testing the questionnaire, EFA, and adjustment of questions, the measurement model in this study consisted of a total of 33 questions, which were used to measure five variables, including cultural identity, travel experience, place attachment, tourist satisfaction, and revisit intention. The measurement model in this study was reflective. CFA was performed using the PLS algorithm of SmartPLS to evaluate its convergent and discriminant validity.

Cultural identity is a second-order variable of different dimensions, which requires a hierarchical CFA. In other words, the first-order structure of the cultural identity measurement model was tested first, and then the second-order structure was tested. The results showed that the Cronbach’s *α* values of the three dimensions of cultural identity were all greater than the standard value of 0.7 (Cronbach, 1951); the *CR* value was above 0.7 and below 0.95, which was an ideal index value, indicating that the first-order structure of the measurement model enjoyed good reliability (Nunnally, 1994). The loadings of the questions in the three dimensions of cultural identity were all greater than 0.7, and the *t* value was at a significant level, suggesting that the questions of the measurement model were reliable. The *AVE* value was greater than the standard value of 0.5, showing that the convergent validity of the first-order structure of cultural identity was good. The Cronbach’s *α* values of the second-order structure of the measurement model were all greater than the standard value of 0.7, and the *CR* value was above 0.7 and lower than 0.95, which was an ideal index value, indicating that the second-order structure of the measurement model was reliable. The dimensional factor loadings of the second-order structure of cultural identity were all above 0.7, and the *t* value was at a significant level, suggesting that the sub-dimensions of the measurement model were reliable (Hair et al., 2017). The *AVE* value was also greater than the standard value of 0.5, indicating that the second-order structure of cultural identity enjoyed good convergent validity (Chin and Newsted, 1999). Subsequently, the same operation was performed on the second-order variables of travel experience and place attachment, and the reliability and convergent validity of tourist satisfaction and revisit intention were evaluated. The results revealed that the first-order and second-order structures of travel experience and place attachment had good reliability, and the first-order and second-order structures boasted sound convergent validity; tourist satisfaction and revisit intention also enjoyed excellent reliability and convergent validity.

The Fornell-Larcker index and HTMT index were mainly used to test the discriminant validity of the partial least squares structural equation model (PLS-SEM). The Fornell-Larcker discriminant validity of the first-order and second-order measurement model was evaluated by Smart-PLS, which demonstrated that the square root of the average extracted variation of all dimensions was greater than the correlation coefficient between this dimension and other dimensions, meeting the discriminant validity evaluation criteria (Fornell and Larcker, 1981). After that, the first-order measurement model and the second-order measurement model were, respectively, evaluated for HTMT discriminant validity, which exhibited that the HTMT indexes were all less than the standard value of 0.9, meeting the requirements of HTMT discriminant validity (Hair et al., 2017).

### 4.2. Structural model evaluation

The structural model was evaluated in the following four steps. *Step 1*: the collinearity of the structural model was evaluated; *Step 2*: the path coefficient *β* and the corresponding *t* values were calculated, where the path coefficient *β* represents the strength of the relationship between dimensions, and the *t* value is used to determine whether the relationship between the dimensions is significant; *Step 3*: the determination coefficient *R^2^* was calculated to measure the predictive accuracy of the external variable to the internal dependent variable; *Step 4*: the predictive correlation *Q^2^* was calculated to measure the predictive correlation of the potential factor in the path model for the dimension.

#### 4.2.1. Collinearity evaluation

The PLS algorithm is easily disturbed by the collinearity, so it is necessary to evaluate the collinearity of the structural model. The collinearity is generally measured by two indicators: tolerance and variance inflation factor (VIF). VIF is the inverse of tolerance (1/tolerance). In PLS-SEM, if VIF is greater than or equal to 5, it means that there may be collinearity (Bricker and Kerstetter, 2000). The PLS algorithm was performed in SmartPLS to calculate the VIF value (Table 3). The VIF value of the structural model was between 1 and 2.561, indicating that the structural model of this study did not have serious collinearity.

**Table 3:** The collinearity evaluation results of the structural model.

	Cultural identity	Travel experience	Place attachment	Tourist satisfaction	Revisit intention
Cultural identity		1.000	2.253	2.595	
Travel experience			2.253	2.460	2.561
Place attachment				1.947	1.736
Tourist satisfaction					2.170

#### 4.2.2. Path coefficient evaluation

The path coefficient *β* is used to determine whether the research hypothesis holds. In this study, the 5,000-sample bootstrapping method was used to calculate the path coefficient using SmartPLS. In the two-tailed test, the *t* value of 1.96 was used as the critical value for the significance level equal to 0.05 (Bricker and Kerstetter, 2000). The path coefficients of the structural model are shown in Table 4.

**Table 4:** The path analysis and hypothesis evaluation results.

	*β*	*t*	*p*	Support hypotheses?
Cultural identity → travel experience	0.746	26.005	0.0000	Yes
Cultural identity → place attachment	0.419	7.481	0.0000	Yes
Cultural identity → tourist satisfaction	0.105	1.863	0.0630	No
Travel experience → place attachment	0.326	5.893	0.0000	Yes
Travel experience → tourist satisfaction	0.576	10.081	0.0000	Yes
Travel experience → revisit intention	0.329	5.659	0.0000	Yes
Place attachment → tourist satisfaction	0.112	2.138	0.0330	Yes
Place attachment → revisit intention	0.288	6.561	0.0000	Yes
Tourist satisfaction → revisit intention	0.257	4.864	0.0000	Yes

Cultural identity had a significant positive impact on travel experience (*β* = 0.746, *t* = 26.005, *p* < 0.001), supporting the research hypothesis H1. Cultural identity is positively associated with place attachment (*β* = 0.419, *t* = 7.481, *p* < 0.001), supporting the research hypothesis H2. Cultural identity is positively associated with tourist satisfaction (*β* = 0.105, *t* = 1.863, *p* = 0.063), not supporting the research hypothesis H3. Travel experience is positively associated with place attachment (*β* = 0.326, *t* = 5.893, *p* < 0.001), supporting the research hypothesis H4. Travel experience is positively associated with tourist satisfaction (*β* = 0.576, *t* = 10.081, *p* < 0.001), supporting the research hypothesis H5. Travel experience is positively associated with revisit intention (*β* = 0.329, *t* = 5.659, *p* < 0.001), supporting the research hypothesis H6. Place attachment is positively associated with tourist satisfaction (*β* = 0.112, *t* = 2.138, *p* < 0.05), supporting the research hypothesis H7. Place attachment is positively associated with revisit intention (*β* = 0.288, *t* = 6.561, *p* < 0.001), supporting the research hypothesis H8. Tourist satisfaction is positively associated with revisit intention (*β* = 0.257, *t* = 4.864, *p* < 0.001), supporting the research hypothesis H9.

#### 4.2.3. Determination coefficient evaluation

The determination coefficient *R^2^* is a measure of the explanatory power of a model (Shmueli and Koppius, 2011). Its value is between 0 and 1; the higher the value, the stronger the predictive power. As a guideline, *R^2^* values of 0.75, 0.50, and 0.25 can be considered to have substantial, moderate, and weak explanatory power (Henseler et al., 2009). Table 5 presents the determination coefficients for correlated variables, with *R^2^* ranging from 0.484 to 0.58, showing moderate explanatory power.

**Table 5:** The explanatory power and predictive correlation evaluation.

	*R^2^*	Adjusted *R^2^*	SS0	SSE	*R^2^* (=1−SSE/SS0)
Travel experience	0.556	0.555	5,532	4118.344	0.256
Place attachment	0.486	0.484	2,766	1946.692	0.296
Tourist satisfaction	0.543	0.54	1,383	849.645	0.386
Revisit intention	0.583	0.58	1,383	820.664	0.407

#### 4.2.4. Predictive correlation evaluation

Another way to evaluate the predictive accuracy of the PLS structural model is to calculate the predictive correlation coefficient *Q^2^* (Geisser, 1974). This value is calculated using the Blindfolding Procedure. *Q^2^* equal to zero indicates that the latent variable is replaced by the mean with no difference in dimensions, and *Q^2^* less than 0 indicates a lack of predictive correlation. Empirically, *Q^2^* values greater than 0, 0.25, and 0.5 indicate minor, medium, and significant predictive correlations for the structural model, respectively (Rigdon, 2014). The predictive correlation evaluation results for the structural model in this study are shown in Table 5, showing that the predictive power is above medium.

In summary, based on the results of collinearity, path coefficient *β*, determination coefficient *R*, and predictive correlation coefficient *Q^2^*, the proposed structural model proposed in this study was considered able to explain the relationship between the dimensions and effectively verify the research hypothesis.

#### 4.2.5. Mediating effect evaluation

In this study, Bootstrapping analysis in Smartpls 3.3.3 was used to evaluate the significance of the mediating effect of dimensions. Bootstrapping is a non-parametric resampling procedure that does not require the assumption of a normal distribution (Preacher and Leonardelli, 2001). In essence, it simulates the random sampling from the total population, which can be used to directly test the mediation effect (Fang and Wen, 2014). The results are shown in Table 6, with 11 significant paths and five approximately significant paths. The study validated the following paths: cultural identity → travel experience → tourist satisfaction → revisit intention; cultural identity → place attachment → tourist satisfaction → revisit intention; cultural identity → travel experience → place attachment → revisit intention.

**Table 6:** Bootstrapping-specific mediating effect analysis.

Path*	Initial sample	Sample mean	SD	*t*	*p*
C → P → R	0.121	0.121	0.027	4.529	0.0000
C → S → R	0.027	0.028	0.016	1.637	0.102
C → P → S	0.047	0.046	0.022	2.098	0.036
E → S → R	0.148	0.15	0.036	4.133	0.0000
E → P → R	0.094	0.094	0.02	4.643	0.0000
C → P → S → R	0.012	0.012	0.006	2.04	0.041
C → E → P → S → R	0.007	0.007	0.004	1.837	0.066
C → E → S	0.43	0.43	0.047	9.097	0.0000
C → E → R	0.245	0.244	0.047	5.239	0.0000
E → P → S	0.037	0.036	0.019	1.943	0.052
P → S → R	0.029	0.028	0.014	2.055	0.04
C → E → P → R	0.07	0.07	0.015	4.556	0.0000
E → P → S → R	0.009	0.009	0.005	1.858	0.063
C → E → P	0.243	0.243	0.044	5.551	0.0000
C → E → S → R	0.11	0.112	0.027	4.047	0.0000
C → E → P → S	0.027	0.027	0.014	1.922	0.055

*C: cultural identity; E: travel experience; P: place attachment; S: tourist satisfaction; R: revisit intention.

## 5. Conclusion

This study constructs a theoretical model for tourists of the Archaeological Ruins of Liangzhu City based on the relevant theories of marketing, psychology, and tourism management, which is used to explore the relationship and interaction between travel experience, place attachment, tourist satisfaction, and revisit intention. The results are obtained through qualitative in-depth interviews and the quantitative structural equation model. The following conclusions are reached.

This study clarifies the concept and dimensions of cultural identity among heritage tourists and establishes a scale that can effectively measure the cultural identity of these tourists. By testing the reliability and validity subdimensions used in previous studies, this study classifies cultural identity into three dimensions: cognitive, emotional, and behavioral. It extracts relevant questions from similar measurement scales and constructs the cultural identity scale of heritage tourists in this study through item analysis.

Cultural identity is positively associated with travel experience and place attachment but not significant associated with tourist satisfaction. The stronger the cultural identity with the destination, the higher the perceived value of the destination and the more positive travel experience. Tourists with a stronger cultural identity are more likely to develop a sense of attachment and belonging to a tourist destination. However, the empirical results of this study show that the cultural identity of heritage tourists does not significantly affect tourist satisfaction. The possible reason is that the direct impact of cultural identity on tourist satisfaction is limited, which does not reach statistical significance, but it affects tourist satisfaction through mediating variables such as travel experience and place attachment.

Place attachment, travel experience, and tourist satisfaction all are positively associated with revisit intention. The structural equation model results demonstrate that place attachment, travel experience, and tourist satisfaction are positively associated with revisit intention. The results of this study show that a good tourism experience can better meet the needs of tourists, encouraging them to revisit and recommend the destination to others; tourists who have a higher level of place attachment through tourism are more likely to have a higher revisit intention; the higher the tourist satisfaction, the stronger the intention to revisit, and the more likely to have revisit and recommendation behavior.

Tourism experience plays a key role in cultural identity forming revisit intention. The structural equation model results exhibit that place attachment, tourism experience, and tourist satisfaction all are positive associated with revisit intention. However, by comparing the path coefficients, it is not difficult to find that the relationship of tourism experience and revisit intention is stronger. Therefore, improving the tourists’ experience is the key link to promoting their intentions to revisit.

**Figure 2:**
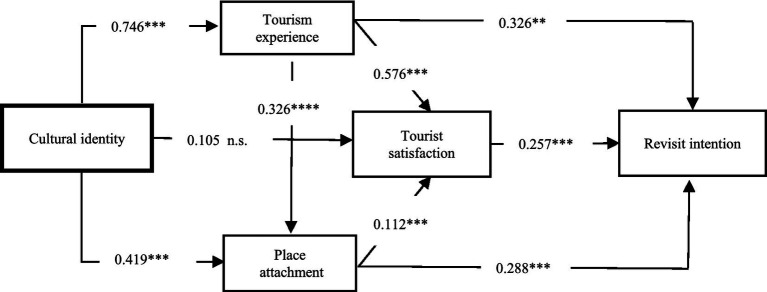
Final model with results. ****p value < 0.0001;***p value < 0.001; **p value < 0.01; *p value < 0.05; n.s.: not significant.

This study verifies the effective way between cultural identity and revisit intention. In this study, the mediating role of travel experience, place attachment, and tourist satisfaction in the research model is analyzed. The present study validates the following paths: cultural identity → travel experience → tourist satisfaction → revisit intention; cultural identity → place attachment → tourist satisfaction → revisit intention; cultural identity → travel experience → place attachment → revisit intention (Figure 2).

## 6. Significance and implications

### 6.1. Theoretical significance

The theoretical contributions of this study are as follows: (1) this study constructs and verifies the cultural identity scale of heritage tourists by taking the Archaeological Ruins of Liangzhu City as an example. (2) it constructs a theoretical model of cultural identity, tourism experience, place attachment, tourist satisfaction, and revisit intention among heritage tourists, puts forward corresponding research hypotheses, and verifies the theoretical relationship between the dimensions. (3) in the context of cultural heritage tourism, the study of tourists’ cultural identity as an independent variable reveals the effective path by which cultural identity towards revisit intention through tourism experience, place attachment, and satisfaction, enriching the research in this field. (4) this study combines qualitative and quantitative analysis for triangulation, complementation, and development of the results.

### 6.2. Management implications

The management implications of this study are as follows. (1) it is necessary to strengthen public education and media publicity, enhance the cultural identity of potential heritage tourists, stimulate the potential tourism demand of the public, and generate good word-of-mouth effects and social benefits. (2) for tourists with different cultural identities, menu-type tour routes can be provided to accurately improve the experience of heritage tourists, improve their satisfaction, and promote their willingness to revisit. (3) it is essential to pay attention to the emotional connection between tourists and tourist destinations, carry out emotional marketing, provide warm services, improve the level of place attachment of tourists, and make them become return customers and promoters of tourist destinations. (4) cultural heritage tourism destinations should strengthen the protection and sustainable development of cultural heritage while encouraging tourists to revisit and demonstrating China’s soft power and cultural confidence.

### 6.3. Limitations

There are still some limitations in this study, which require further inquiry. (1) this study did not make any distinction in the analysis of the sample data of heritage tourists. In the follow-up research, it may be necessary to classify tourists according to the source of tourists, frequency of visits, etc. On this basis, group analysis can be conducted to test the model constructed in the present study and examine the impact of the categorical variable on the strength of the relationship between the dimensions. (2) the case selected in this study is the Archaeological Ruins of Liangzhu City. In the future, other cultural heritages can be selected to further verify the research model established in this study. (3) this research is mainly based on quantitative analysis. In the future, qualitative analysis can be carried out based on in-depth interviews with cultural heritage managers and tourists, thereby providing more practical recommendations.

## Data availability statement

The datasets presented in this study can be found in online repositories. The names of the repository/repositories and accession number(s) can be found in the article/Supplementary material.

## Ethics statement

Ethical review and approval were not required for the study on human participants in accordance with the local legislation and institutional requirements. Written informed consent from the [patients/participants OR patients/participants legal guardian/next of kin] was not required to participate in this study in accordance with the national legislation and the institutional requirements.

## Author contributions

JL: conceptualization, supervision, and funding acquisition. YF: methodology, validation, formal analysis, data curation, writing—original draft preparation, and writing—review and editing. All authors contributed to the article and approved the submitted version.

## Funding

This research was funded by the General Research Project of Zhejiang Provincial Department of Education, grant number Y202045028.

## Conflict of interest

The authors declare that the research was conducted in the absence of any commercial or financial relationships that could be construed as a potential conflict of interest.

## Publisher’s note

All claims expressed in this article are solely those of the authors and do not necessarily represent those of their affiliated organizations, or those of the publisher, the editors and the reviewers. Any product that may be evaluated in this article, or claim that may be made by its manufacturer, is not guaranteed or endorsed by the publisher.
